# PsyMuKB: An Integrative *De Novo* Variant Knowledge Base for Developmental Disorders

**DOI:** 10.1016/j.gpb.2019.10.002

**Published:** 2019-12-04

**Authors:** Guan Ning Lin, Sijia Guo, Xian Tan, Weidi Wang, Wei Qian, Weichen Song, Jingru Wang, Shunying Yu, Zhen Wang, Donghong Cui, Han Wang

**Affiliations:** 1 Shanghai Mental Health Center, Shanghai Jiao Tong University School of Medicine, School of Biomedical Engineering, Shanghai Jiao Tong University, Shanghai 200030, China; 2 Shanghai Key Laboratory of Psychotic Disorders, Shanghai 200030, China; 3 Brain Science and Technology Research Center, Shanghai Jiao Tong University, Shanghai 200030, China; 4 College of Information Science and Technology, Northeast Normal University, Changchun 130117, China; 5 Institution of Computational Biology, Northeast Normal University, Changchun 130117, China

**Keywords:** *De novo* mutation, Developmental and neuropsychiatric disorders, Database, Isoforms, Tissue specificity

## Abstract

*De novo* variants (DNVs) are one of the most significant contributors to severe early-onset genetic disorders such as autism spectrum disorder, intellectual disability, and other developmental and neuropsychiatric (DNP) disorders. Presently, a plethora of DNVs have been identified using next-generation sequencing, and many efforts have been made to understand their impact at the gene level. However, there has been little exploration of the effects at the isoform level. The brain contains a high level of alternative splicing and regulation, and exhibits a more divergent splicing program than other tissues. Therefore, it is crucial to explore variants at the transcriptional regulation level to better interpret the mechanisms underlying DNP disorders. To facilitate a better usage and improve the isoform-level interpretation of variants, we developed NeuroPsychiatric Mutation Knowledge Base (PsyMuKB). It contains a comprehensive, carefully curated list of DNVs with transcriptional and translational annotations to enable identification of isoform-specific mutations. PsyMuKB allows a flexible search of genes or variants and provides both table-based descriptions and associated visualizations, such as expression, transcript genomic structures, protein interactions, and the mutation sites mapped on the protein structures. It also provides an easy-to-use web interface, allowing users to rapidly visualize the locations and characteristics of mutations and the expression patterns of the impacted genes and **isoforms**. PsyMuKB thus constitutes a valuable resource for identifying tissue-specific DNVs for further functional studies of related disorders. PsyMuKB is freely accessible at http://psymukb.net.

## Introduction

In addition to inheriting half of each parent’s genome, each individual is born with a small set of novel genetic changes, referred to as *de novo* variants (DNVs), that occur during gametogenesis [1], [2]. These variants are identified in parent-offspring trios and implicated in various human diseases [3], [4]. Among DNVs, *de novo* mutations (DNMs) are those variants range in size from single-nucleotide variants (SNVs) to small insertions and deletions (indels). In contrast, larger structural variations in DNVs are known as *de novo* copy number variations (CNVs). Recently, a large number of DNVs have been identified by whole-exome sequencing (WES) and whole-genome sequencing (WGS), and have been explored and analyzed at the gene level to assess their contributions to complex diseases [5], [6], [7], [8], [9], [10]. However, isoform level information has rarely been explored.

As many as 95% of genes are subject to alternative splicing, initiation, and promoter usage to produce various isoforms, increasing human transcriptomic and proteomic diversity [11], [12], with approximately four to seven isoforms per gene [12], [13]. Some isoforms can be highly specific, and their expressions are often restricted to certain organs, tissues, or even cell types within the same tissue [14], [15], [16]. Notably, this occurs at a high frequency in brain tissues [17], [18] and regulates biological processes during neural development, including cell-fate decisions, neuronal migration, axon guidance, and synaptogenesis [19], [20].

Exons can be differentially used in isoforms of the same gene, and disease mutations may selectively impact only isoforms with mutation-carrying exons. Moreover, if some isoforms are not expressed in a particular developmental period or a specific tissue, then disease mutations affecting such isoforms may not manifest their functional impact at that period or in that tissue. Thus, correlating tissue-specific isoforms with disease mutations is an important and necessary task for refining our understanding of human diseases. Since the brain is subject to a high number of alternative splicing events [17], [18], it is imperative that mutations related to brain disorders, with brain-specific expression, are studied at the isoform level. However, the association between isoforms and DNMs has rarely been investigated on a large scale in developmental and neuropsychiatric (DNP) disorders, such as autism (ASD), schizophrenia (SCZ), early-onset Alzheimer’s disease (AD), and congenital heart defect (CHD).

**Figure 1:**
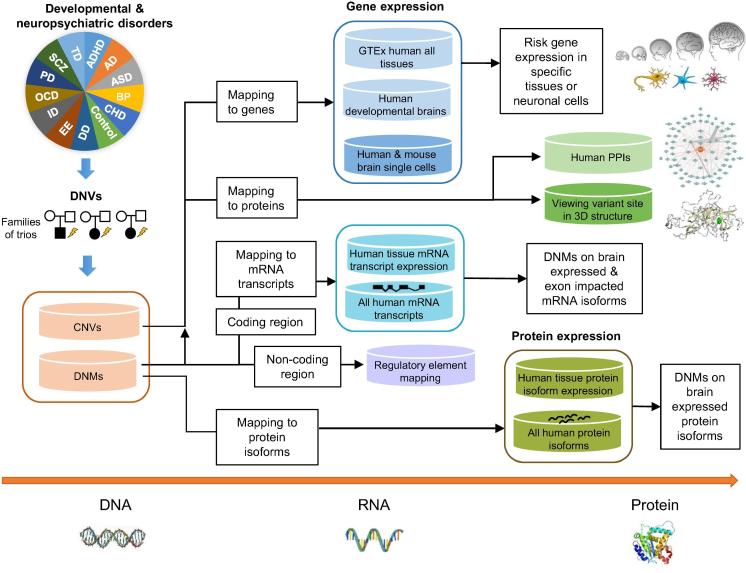
**Flowchart of PsyMuKB** PsyMuKB is a multi-level DNV exploration knowledge base integrating various transcriptional and translational annotations to aid the understanding of the genetic variants from DNP disorders. Variants and genes carrying variants can be browsed, searched and filtered at gene, protein, mRNA isoform, or protein isoform levels with related annotations, such as tissue and neuronal cell-specific expression patterns, protein interactions, mutation sites mapping to regulatory elements, and protein 3D structures by muPIT [40], *etc*. The isoform-specific mutation filtering, using tissue-specific expression patterns and isoform exon–intron structure information, is one of the key functionalities in PsyMuKB. CNV, copy number variation; DNM, *de novo* mutation; DNV, *de novo* variant; PPI, protein–protein interaction; AD, Alzheimer’s disorder; ADHD, attention deficit hyperactivity disorder; ASD, autism spectrum disorder; BP, bipolar disorder; CHD, congenital heart defect; DD, developmental delay; EE, epileptic encephalopathies; ID, intellectual disability; OCD, obsessive–compulsive disorder; PD, Parkinson’s disease; SCZ, schizophrenia; TD, Tourette disorder.

In this study, we present the NeuroPsychiatric Mutation Knowledge Base (PsyMuKB), a unique DNV database that we have developed. PsyMuKB serves as an integrative platform that enables exploration of the association between tissue-specific regulation and DNVs in DNP disorders (Figure 1). It provides a comprehensive collection of DNVs reported in 123 studies as of May 2019: both DNMs hitting coding and non-coding regions and *de novo* CNVs, spanning across 25 different clinical phenotypes, such as ASD, SCZ, and early-onset AD. In addition, we developed a novel pipeline that allows flexible filtering and exploration of isoforms that are impacted by mutation and/or brain-expressed with a user-specified selection, based on the genomic position of each mutation, transcriptional features, and the genomic structures of transcripts. Finally, PsyMuKB allows the searching and browsing of genes by their IDs, symbols, or genomic coordinates, and provides detailed gene information, including descriptions and summaries, exon–intron structures of transcripts, expression of the gene and/or protein in various tissues, and protein–protein interactions (PPIs). Therefore, PsyMuKB is a comprehensive resource for exploring disease risk factors by transcriptional and translational information with associated visualizations. Herein, we describe the architectural features of PsyMuKB, including both the variants and their annotations, and a system for understanding the impact of mutations on tissue-specific isoforms in brain-related complex disorders. It highlights novel mechanisms underlying the genetic basis of DNP disorders.

## Data collection and processing

### DNV curation

PsyMuKB catalogs two types of DNVs: (1) DNMs that include *de novo* point mutations and small indels; (2) *de novo* CNVs that involve deletions or duplications in copy numbers of specific regions of DNA. We first surveyed the literature for all published studies where human DNVs had been identified at a genome-wide scale [21]. All studies were then carefully curated to maintain essential information on each DNV, including sample identifier (if available), chromosomal locations of the reference and alternative alleles, validation status. All variants’ coordinates are shown in GRCh37 (hg19) in PsyMuKB for both DNMs and *de novo* CNVs. If source variant coordinates were not originally provided in GRCh37, the coordinates were then lifted-over using the “LiftOver” from the UCSC genome browser (http://genome.ucsc.edu/cgi-bin/hgLiftOver) for annotation consistency.

The vast majority of DNM studies published and included in PsyMuKB have employed large-scale parallel sequencing using mostly WES but sometimes WGS, in conjunction with large sample sizes (hundreds to thousands of samples). These were collected mostly from family trios, but sometimes family quads [21]. By comparing the DNA sequences obtained from affected children to those from their parents, it is possible to identify DNMs after filtering out sequencing artifacts and variant-calling errors. The variant-calling process requires a detailed bioinformatics pipeline involving the application of different thresholds to filter for various quality parameters, such as allele balance (*e.g.*, AB between 0.3 and 0.7), allele depth (*e.g.*, DP ≥ 20), genotype quality (*e.g.*, GQ ≥ 20), mapping quality (*e.g.*, MQ ≥ 30), allele frequency in general population (usually < 1% or 0.1% as a more stringent cutoff), *etc*. [5], [22]. Nonetheless, all DNMs (or randomly selected subsets) are re-sequenced by other methods, usually Sanger sequencing, to check the accuracy of the findings. As a result, the average rate of DNM is estimated to be 1–3 per individual in the whole exome and 60–80 per individual in the whole genome [23]. During our data collection and curation process, we ensured all the DNM data included in PsyMuKB that came from discovery pipelines with reasonable quality parameters, such as those used in the 2018 study by Werling et al. [5]. Next, all the collected DNMs were batch-processed for systematic annotations using the ANNOVAR annotation platform [24] to include annotations, such as variant function (exonic, intronic, intergenic, UTR, *etc*.), exonic variant function (non-synonymous, synonymous, *etc*.), amino acid changes, frequency in the 1000 genome and ExAC database [25], and variant functional predictions by SIFT [26], Polyphen2 [27], GERP++ [28], and CADD [29]. Since the emphasis of many available functional annotations of variants is on coding regions, we included the DeepSea scores in the variant annotation table to help users evaluate the impact of the variants at non-coding locations. In addition, for each gene, we included the Haploinsufficiency Score [30] for assessing the likelihood of the gene exhibiting haploinsufficiency and the probability of being loss of function (LoF) intolerant (pLI) score [25] for assessing the probability of it being intolerant to LoF variants.

### Collection and processing of expression datasets

PsyMuKB currently includes five different datasets for expression annotations, of which four datasets are transcriptomic data, and the last one is protein expression data. We selected four large-scale transcriptomic study datasets to comprehensively annotate and illustrate transcriptional expression, including human tissue expressions from the Genotype-Tissue Expression (GTEx) consortium [31] (http://www.gtexportal.org/home), the BrainSpan Atlas of the Developing Human Brain [32] (www.brainspan.org), and human embryonic prefrontal cortex single-cell expressions [33]. Considering the majority of developmental regulation modules are preserved between human and mouse [34], we also integrated adult mouse brain single-cell expression atlas data (DropViz: http://dropviz.org) [35], to expand the interpretive annotations of genes associated with DNVs. Gene expression levels were summarized as either reads per kilobase million (RPKM), or transcripts per million (TPM) as provided by their respective sources. We calculated and visualized all the expression levels by either original or Log_10_ based normalized values. In PsyMuKB, the BrainSpan data are displayed across six brain regions and nine developmental periods, while GTEx data are displayed by listing all human tissues in alphabetical order. All neuronal cell types are annotated by their major cell types, such as neuron, interneuron, microglia, stem cell, oligodendrocyte progenitor cell (OPC), astrocyte, *etc*. The human brain single-cell expressions are visualized by developmental periods and cell types, while the mouse brain single-cell expressions are visualized by brain regions and cell types. These gene expression patterns mainly aid exploration of the role of a gene in normal tissues or developmental periods, not in abnormal situations. Next, we focused on transcripts where DNMs were mapped to their exon locations, and the brain regions where their expressions were recorded. To associate mutations with the brain-expressed transcripts, we mapped the genomic locations of DNMs to the exon–intron structures of each expressed gene isoform.

To associate the mutations with the protein level annotations, we extracted the protein isoform expression data of various human tissues from ProteomicsDB (http://www.proteomicsdb.org). Protein isoform expression data were directly obtained from ProteomicsDB with median Log_10_ based normalized iBAQ intensities as the expression levels. To associate the mutations with the protein isoforms expressed in the brain, we first mapped the mutation genomic locations to all the Gencode mRNA transcripts. Then, we linked Gencode mRNA transcript IDs and UniProt IDs, which in turn were used to identify protein isoform expression data provided by ProteomicsDB. After this, we mapped the expression data to all proteins and their isoforms by UniProt IDs, and all protein expression information was plotted as histograms by different tissue types, *e.g.*, the brain.

### Regulatory element curation and mutation mapping

**Figure 2:**
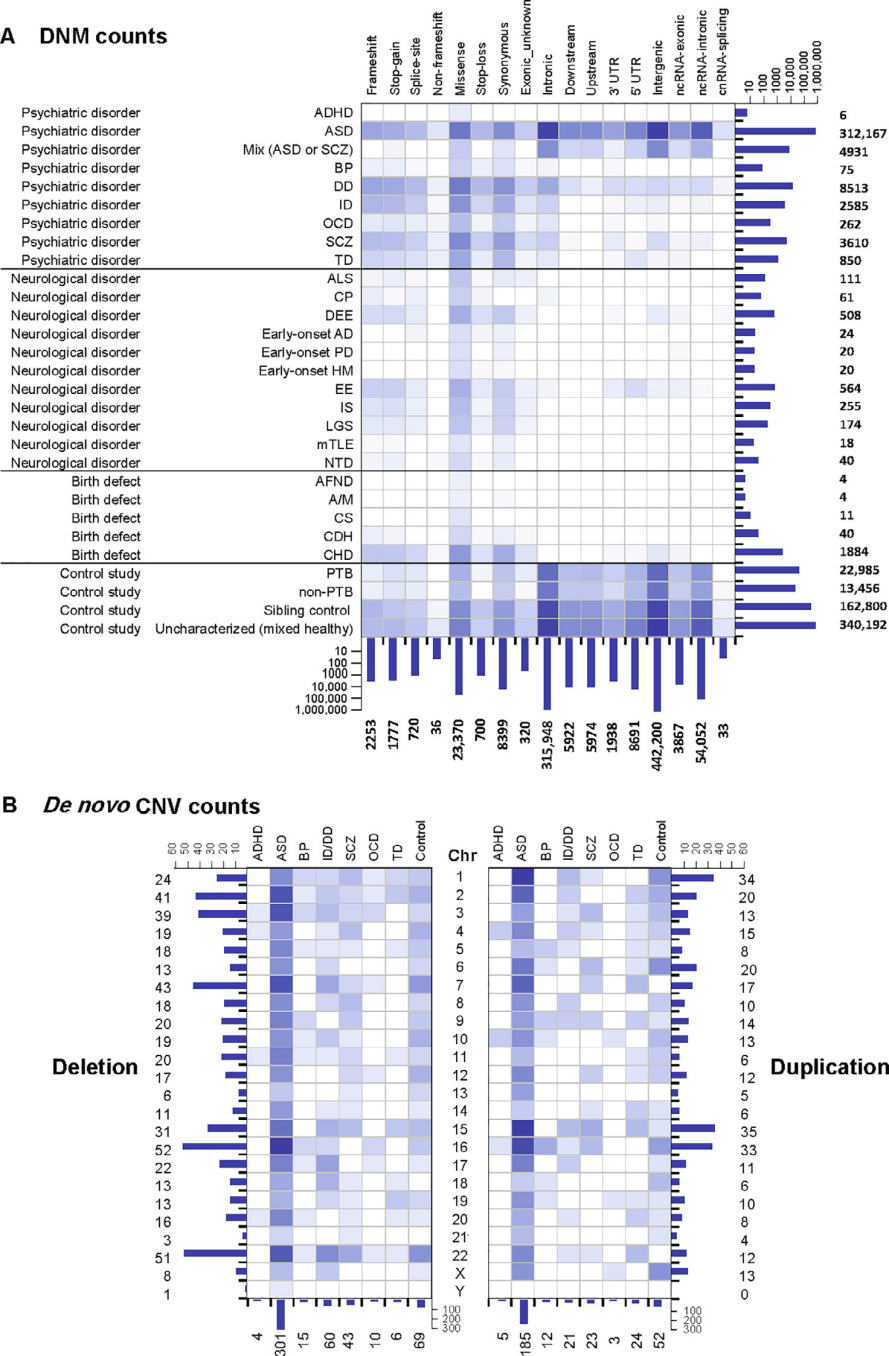
**Statistics of the DNVs collected by PsyMuKB** **A.** The heatmap shows the statistics of DNMs currently in PsyMuKB. The DNMs are divided into four different clinical phenotypes (in rows): psychiatric disorder, neurological disorder, birth defect disorder, and control study. The functional categories of DNMs are separated into 17 different types (in columns), by impacted coding regions (frameshift, stop-gain, spliced-site, non-frameshift, missense, stop-loss, synonymous, and exonic unknown), to the non-coding areas (intronic, downstream, upstream, 5′-UTR, 3′-UTR, intergenic, and ncRNA). **B.** The heatmap shows the statistics of *de novo* CNVs currently in PsyMuKB. CNVs are collected from nine different clinical phenotypes, including ADHD, ASD, and SCZ (in columns). The figure is separated into two panels. The left represents deletion events in CNVs, and the right panel represents the duplication events in CNVs. The color in the heatmap represents the counts of each phenotype for a particular variation type with the darker the color, the higher the count. ALS, amyotrophic lateral sclerosis; CP, cerebral palsy; DEE, developmental and epileptic encephalopathies; HM, high myopia; IS, infantile spasms; LGS, Lennox Gastaut syndrome; mTLE, mesial temporal lobe epilepsy; NTD, neural tube defect; AFND, acromelic frontonasal dysostosis; A/M, anophthalmia and microphthalmia; CS, Cantu syndrome; CDH, congenital diaphragmatic hernia; PTB, fetal preterm birth.

**Figure 3:**
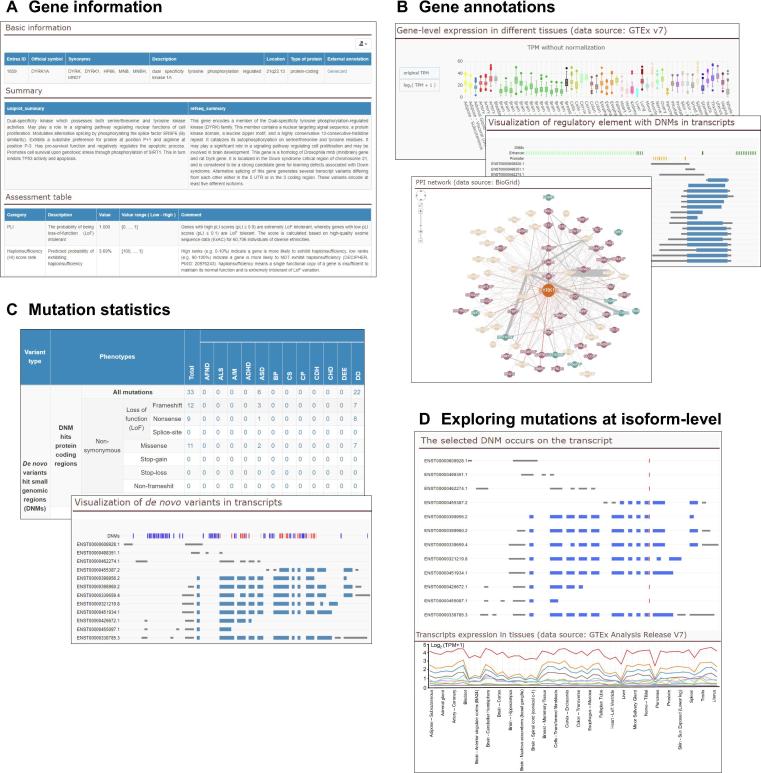
**Web interface of PsyMuKB** A snapshot of the major functionalities of PsyMuKB using neuropsychiatric-related gene *DYRK1A* as an example**. A.** General information about *DYRK1A*, including description, summaries, and gene pathogenicity assessment. **B.** The information about the expression, associated regulated element, PPIs of *DYRK1A*. **C.** The mutation summary table of *DYRK1A* and the visualization of genomic locations of mutations in different transcripts. **D.** The interface for identifying the isoform-specific mutations of *DYRK1A* using the tissue-specific expression of isoforms and their exon–intron structure map. PPI, protein–protein interaction.

Currently, functional annotations mostly emphasize mutations in coding regions. However, more than 90% of all the reported DNMs locate in non-coding regions of the genome (Figure 2**A**). And unlike for coding regions, there is no clear hypothesis of which non-coding areas harbor disease-causing rare variants in humans, nor is it understood which specific alleles are intolerant to a mutation within those non-coding regions. To facilitate the usage of these variants and better explore the potential impact of the mutations hitting the non-translated genomic regions, PsyMuKB provides regulatory element annotations to help investigate whether a non-coding mutation hits a regulatory element, and then potentially influencing downstream gene/isoform targets. This information is in the “Transcripts” subsection of the “Gene Information” page. There are 250,733 gene enhancer regions defined by GeneHancer [36] and 82,149 promoters defined in phase 2 of FANTOM5 [37]. We mapped the curated DNMs that are located in non-coding areas of the genome to all the regulatory regions and listed them as part of the mutation annotations (Figure 3).

### Interaction data curation

We extracted PPI data from BioGRID [38] to construct a comprehensive map of physically interacted human proteins. After removing non-physical interactions as defined in BioGRID, we obtained 409,173 human PPIs for annotation integration, allowing users to explore the potential functional pathways involving the proteins impacted. For each interaction, we kept the annotations, such as official symbols of both protein interactors, experiment detection methods, and publication PMIDs.

### Database architecture

PsyMuKB has been designed as an expandable big data platform using MongoDB, a high-performance non-SQL database management system. This design provides PsyMuKB with sufficient scalability and extensibility for easy and fast data integration and module expansion in future updates. All metadata in PsyMuKB are stored in the MongoDB database, while the graphical representations, such as expression profiles, mutations mapping to the transcripts, and PPI network, are mapped and drawn in real-time when related data are queried. The web interface and data visualization of PsyMuKB was implemented mostly in Python scripts based on HTML5 and Cascading Style Sheets (CSS), and JavaScript (JS). The expression data visualization and regulatory element mapping were implemented using Plotly. The interaction network visualization was implemented using Cytoscape.js [39]. Illustration of the mutation site in a 3D protein structure is provided by a link to the corresponding visualization provided by the muPIT [40] interactive web server (http://mupit.icm.jhu.edu/MuPIT_Interactive).

## Database content and usage

### Mutation data statistics

As of May 2019, PsyMuKB contains 877,019 DNVs in the current PsyMuKB.v.1.5 version, covering 24 different types of brain or neuronal-related disorders and some control population studies (Figure 2A, Data collection and processing). A total of 876,178 of them are DNMs, including SNVs and small indels, affecting 732,879 unique sites across the genome (Figure 2A). About 61.5% of variants come from controls, including healthy sibling of patients (*n* = 162,800) from various DNP disorder studies, an uncharacterized cohort study (*n* = 340,192) [41], and a fetal sample (preterm and non-preterm) study (*n* = 36,441) [42]. DNM variants were collected from various studies based on four major clinical phenotypes: psychiatric disorders, neurological disorders, birth defect diseases, and control studies (Figure 2A). In eight major developmental psychiatric disorders, the majority (93.7%) of DNMs came from ASD studies (*n* = 312,167), followed by studies of developmental delay (DD) (*n* = 8513), SCZ (*n* = 3610) and intellectual disability (ID) (*n* = 2585). In neurological disorders, the majority of DNMs came from epileptic encephalopathies (EE) (*n* = 564), and developmental and epileptic encephalopathies (DEE) (*n* = 508). In birth defect diseases, the majority of DNMs came from CHD (97%, *n* = 1884). For DNMs, half of the variants were located in intergenic regions (*n* = 442,200), compared to only about 4.3% (*n* = 28,259) of mutations impacting exonic regions and 38.7% located at 5′-UTR, 3′-UTR, intronic, upstream or downstream regions of the transcripts, while the remaining 6.6% of DNMs were locating in non-coding RNAs (Figure 2A).

It has been shown that CNVs have contributed significantly to the disease etiology of psychiatric disorders [43], [44], [45], [46]. Thus, it is vital that such variants are included in the database as well. Therefore, we curated 841 *de novo* CNVs from reported genome-scale studies, covering eight different clinical phenotypes and affecting 369 non-overlapping genomic regions (Figure 2B, Data collection and processing), ranging from 1 Kb to 600 Mb. More than half of *de novo* CNVs are ASD CNVs (28%, *n* = 486), followed by control (14%), ID (9.6%), and SCZ (7.8%) CNVs. Additionally, we showed that CNVs hit most frequently at regions of chromosome 16 (10%, *n* = 85), followed by chromosomes 22, 2, 7, and 1 (Figure 2B).

### Novelty of PsyMuKB

PsyMuKB does not limit its collection of variants to DNMs like three existing databases, the Developmental Brain Disorder Genes Database (DBD) [47], denovo-db [48], and NPdenovo [49]. It also provides a comprehensive list of *de novo* CNVs covering eight different major phenotypes (Figure 2B, Data collection and processing), including DNP disorders, such as ASD and SCZ. DBD (http://dbd.geisingeradmi.org) focuses on six developmental brain disorders, ASD, ID, attention deficit hyperactivity disorder (ADHD), SCZ, bipolar disorder (BP), and epilepsy. It also presents only a selected set of 29 genes carrying missense DNMs and a set of 465 genes with pathogenic LoF (such as splice-site, stop-gain, and frame-shift) in a tiered classification based on LoF count. In addition, the current version (accessed on version 20190705) does not provide data visualization.

While denovo-db (http://denovo-db.gs.washington.edu/denovo-db) maintains a large set of DNMs (∼420,000 DNMs in denovo-db.v.1.6.1) from a wide range of DNP diseases, it focuses on presenting DNMs as a variant collection with annotations, such as variant locations, types, frequencies, and pathogenicity predictions by SIFT [26], CADD [29], *etc*. In its current version (accessed on version 20190705), it lacks additional genetic annotations such as gene and/or protein expression and PPIs, which would allow further interpretation. In addition, like the DBD database, it does not provide any data visualization in the current version. The third database, NPdenovo (http://www.wzgenomics.cn/NPdenovo), covers ∼97,000 DNMs in both coding and non-coding regions across a selected set of neuropsychiatric disorders, ASD, intellectual disability, SCZ, EE, and controls (accessed on version 20190705), whereas PsyMuKB contains ∼730,000 DNMs across genomes from at least 24 different brain disorders. Both NPdenovo and PsyMuKB include additional genomic and proteomic information for risk assessment, such as gene expression and PPIs. However, PsyMuKB offers interpretations of the variants at mRNA isoform, gene, and protein isoform level in a color interactive visualization, in contrast to the current version of NPdenovo only offering interpretations of gene-level expression data in tabular format.

Thus, our knowledge base differs from the existing databases by assembling DNVs regardless of clinical phenotypes and variant types, integrating with various levels of genomic and proteomic annotations. PsyMuKB is envisioned to be an exploratory interpretation platform of all DNVs at the levels of isoform, gene, and protein.

### PsyMuKB website

The PsyMuKB platform consists of a database and web interface with a set of options that support the searching, filtering, visualizing, and sharing of queried data (Figure 3). The retrieving and visualization of gene-level information in PsyMuKB is achieved in three different ways: by “Gene IDs” or “Gene symbols”, “Chromosomal regions”, and “Variants”. A “Gene IDs” or “Gene symbols” search, provided in both “Basic” and “Advanced” searches, is useful in terms of retrieving gene descriptions and summaries (Figure 3A), expression, protein interaction (Figure 3B), and all reported DNVs (Figure 3C) that are associated with the gene, and the supporting evidence. The “Chromosomal regions” search, also provided in both “Basic” and “Advanced” searches, is useful when a user is interested in retrieving all the genes and variants located within a specific region. In addition, PsyMuKB allows the user to browse through genes in the “Browse” tab by alphabetical order of their official gene symbols. The “Browse” tab also allows the user to navigate through different developmental or neuropsychiatric disorder related gene sets. Once a gene is selected, the results are shown in the same way as through the “Search” option.

When a user makes a gene query, PsyMuKB takes the user to a page with a table displaying all the genes with fully and partially matched IDs or gene symbols. This table provides two clickable links: “Gene Information” and “Mutation Information”. The first one links to the gene information page, which contains five different sub-sections: (1) “Gene information”, which has details including descriptions and function summaries; (2) “Expression”, contains gene and protein expression in different tissues; (3) “*De novo* variant”, provides an overview of available DNVs for the queried gene; (4) “Transcript”, provides genomic structure information for all transcripts of the queried gene; and (5) “Protein-protein interaction”, lists all physical interactions involving the queried gene. In “Gene information” section, PsyMuKB provides an “Assessment Table”, which includes several brain- or disease-related genetic features, such as pLI score, haploinsufficiency score rank, *etc*., to help the user better understand the relationship between the gene and diseases.

DNVs can be accessed via two different approaches: (1) through the *de novo* variants statistic table (Figure 3C) of the gene information page after searching by “Gene ID” or “Gene Symbol”, the table lists all reported variants with a hit in the gene of interest; (2) by specifying chromosomal regions, variants types, and/or clinical phenotypes in the advanced search to narrow the results. The variants are grouped by the genes annotated as associating with them, such as at the exonic, UTR or intronic region of a gene, or regulatory regions of one or multiple genes, or intergenic (in-between) of two distant genes. Thus, if a user queries a gene, all the related variants are shown together in two tables: coding mutations and non-coding mutations. The variant tables include information about the mutation, such as location, mutation type, case or control, disease phenotype, mutation site in the protein structure, validation status, frequency in major population databases (1000 genome, ExAC, gnomAD). Importantly, PsyMuKB provides a “Potential Severity Level” assessment annotation with three severity level defined: (1) high severity: a coding variant is either a LoF mutation or otherwise predicted as pathogenic (or deleterious) by at least three of five widely used pathogenicity prediction tools (SIFT, Polyphen2, GERP++, CADD, and ClinVar); (2) medium severity: a coding variant is predicted as pathogenic (or deleterious) by one or two of five prediction tools; (3) low severity: all other coding variants. This mutation-level assessment, together with the gene-level assessment, aids a greater understanding of the queried gene and the specific mutation carried by it.

PsyMuKB also provides basic genomic information on annotated regulatory elements, such as promoters and enhancers, by visualizing their locations on mRNA transcripts of the queried gene (Figure 3B). Moreover, all reported DNMs are mapped and visualized on top of the exon–intron structure of the mRNA transcripts, together with their regulatory elements, which may aid elucidation of the potential roles of the regulatory elements. In addition, PsyMuKB utilizes alternatively spliced isoforms with tissue-specific expression information, together with DNM mapping on top of the isoform structures, to provide isoform-specific mutation selections (Figure 3D).

PsyMuKB also provides a human protein interaction map for the queried protein (Figure 3B). The interaction network is constructed using both first- and second-degree interactions and interactively visualized using Cytoscape.js [39]. The first-degree interactions are defined as the interactions between all proteins and the queried protein. The second-degree interactions are defined as all the interactions between the interacting protein partners of the queried protein. The line thickness for an interaction represents the number of items of supporting evidence. We defined evidence as either a single reported publication or a single supported experiment. If the number of PPI network protein nodes of the queried protein exceeds 200, the network will only display interactions with at least two evidence items. Besides the visualization, we provide a PPI table, which lists all the interaction information, including experiment detection methods, reported publications and total evidence count, regardless of the amount of evidence.

### Exploring mutations at the isoform-level

**Figure 4:**
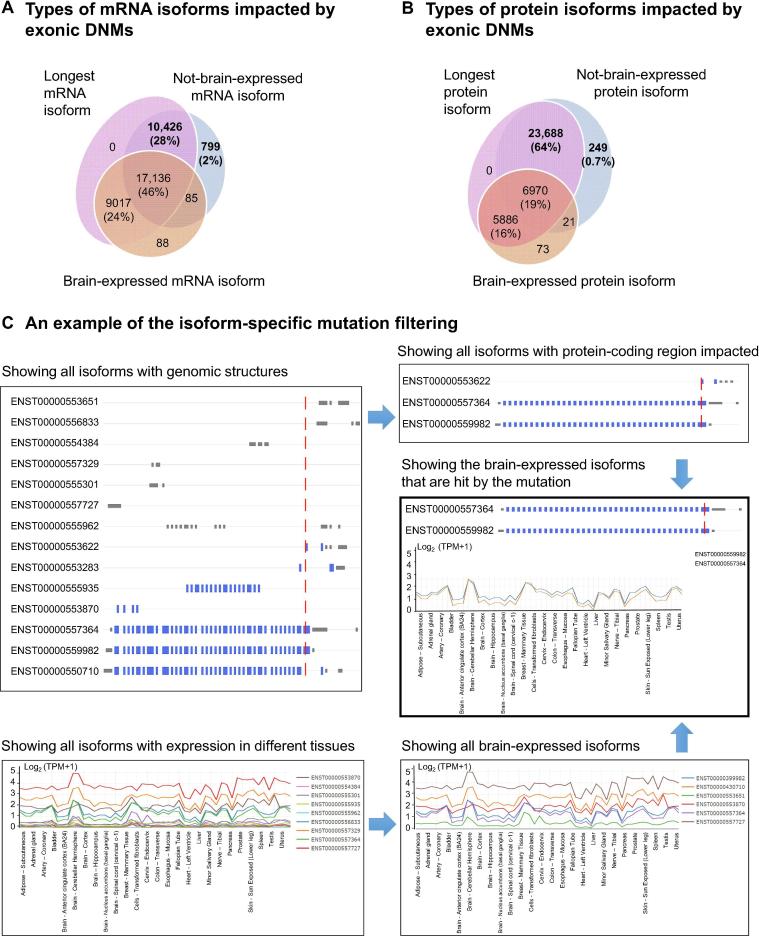
**Exploration of mutations at the isoform-level** **A.** The pie chart shows the overlaps and percentages of DNMs at exonic regions hitting the three different types of mRNA isoforms, illustrating the proportion of DNMs impacting “not-brain-expressed” mRNA isoforms. **B.** The pie chart shows the overlaps and percentages of DNMs at exonic regions hitting the three different types of protein isoforms, illustrating the proportion of DNMs impacting “not-brain-expressed” protein isoforms. **C.** An example of the isoform-specific mutation filtering pipeline using a stop-gain DNM in chromosome 14 at position 21,899,618 with a “G > C” change in gene *CHD8*.

One of the key features of PsyMuKB is that it allows visualization of the DNM locations at the transcript-level and identification of affected isoforms with tissue-specific expression annotations, both at mRNA and protein levels. Here, we first assessed the necessity of studying DNMs at the isoform level and explored the scale of the DNMs that satisfy the criteria above. We started by illustrating the importance of isoform specificity in DNMs. We used all mRNA transcripts from Gencode v19 and mapped all the DNMs in coding regions to all transcripts carrying coding exons and calculated the proportion of the DNMs that are isoform-specific. As a result, we observed that about 19% of all DNMs in PsyMuKB are isoform-specific. DNMs from Birth defect carried the highest percentage (21.2%), followed by psychiatric disorder (18.4%), neurological disorder DNMs (16.8%), and control studies (16.8%). Next, we assessed the importance of tissue-specific isoform in DNMs. We used all mRNA transcripts and protein isoforms from UniProtKB (version released on 2018_07), and defined three types of isoforms, “longest isoform”, “brain-expressed isoform” and “not brain-expressed isoform” (Figure 4). At the mRNA level, the “longest isoform” is the isoform with the longest coding sequence compared to all other isoforms of the same gene. The “brain-expressed isoform” is an isoform with expression of TPM ≥ 1 in at least one brain tissue from GTEx data. The “not brain-expressed isoform” is an isoform that is not expressed (TPM < 1) in any brain tissue sample from GTEx data. At the protein level, the “longest isoform” is the isoform with the longest amino acid sequence in a protein. The “brain-expressed isoform” is an isoform with expression of iBAQ intensity ≥ 1 in at least one brain tissue from ProteomicsDB data. The “not brain-expressed isoform” is an isoform that is not expressed (iBAQ intensity < 1) in any brain tissue sample from ProteomicsDB data.

We annotated those DNMs in PsyMuKB hitting brain-expressed isoforms and identified these as “brain-expressed” mutations, as well as identifying “not-brain-expressed” mutations. Although DNMs can occur anywhere in the genome, the exome or protein-coding region of the genome is often investigated first when studying human disease [6], [7], [50]. Therefore, “not-brain-expressed” mutations may not be as interesting to researchers studying tissue-specific disease biology.

Using the “longest isoform” as the reference isoform has been a common practice in many studies and databases. Here, we ask whether the longest isoform strategy is still applicable for studying tissue-specific mutations. First, we looked at the exonic DNMs that impact isoforms and observed that the majority would hit the longest isoforms as expected due to the length: 97% at the mRNA level and 99% at the protein level. However, when checking whether most DNMs would hit at least one brain-expressed isoform, we observed that about 28% of DNMs do not hit any brain-expressed mRNA isoforms (Figure 4A), and as many as 64% of DNMs do not hit any brain-expressed protein isoforms (Figure 4B), based on the current protein isoform annotation and protein expression information from ProteomicsDB. The results show that investigation of the impact of the disease variants at the isoform-level and tissue specificity is imperative. This is a key reason for PsyMuKB to include tissue- and isoform-specific expression for investigating disease-relevant mutations.

To illustrate the exploration of isoform-specific features using PsyMuKB (Figure 3D), we have showcased this functionality with the neuropsychiatric disease associated gene Chromodomain helicase DNA binding protein 8, *CHD8* (Figure 4C), which has multiple alternative spliced isoforms and wide-spread expression across many tissues. *CHD8* is believed to affect the expression of many genes that are involved in prenatal brain development and is a strong risk factor for DNP disorders, such as ASD [51], [52], [53]. Figure 4C demonstrates the isoform-specific filtering process to identify suitable models for the study of mutations in *CHD8*.

## Concluding remarks

PsyMuKB focuses on the exploration and characterization of DNV data with integrative annotations, such as isoforms, expression, protein interactions, and protein structures, and can be accessed through a user-friendly web interface (http://psymukb.net). Unlike existing databases, PsyMuKB has a “Mutation” interface after a gene has been queried or browsed, which allows a unique and useful investigation of mutations with the added complexity of the alternative splicing and brain-specific expression both at mRNA and protein levels. PsyMuKB aims to be the knowledge base that takes into consideration of the isoform specificity in different tissues when exploring variants, as a specific variant could have a differential impact on alternatively spliced and regulated isoforms, both at the transcriptional and translational levels. Consequently, it could induce an incompletely penetrant effect. Notably, the flexibility of PsyMuKB filtering the isoforms impacted by mutation using their genomic features and expression enhances the ability to identify the tissue-specific *de novo* events. In addition, PsyMuKB is an integrative graphical exploration platform containing a comprehensive list of DNVs, together with various types of graphical transcriptional and translational annotations, such as the detailed genomic structures of transcripts, tissue-specific expression in both genes and proteins, PPIs, and pathogenicity assessments. Thus, PsyMuKB is a comprehensive platform aiding the understanding of the impact of DNVs on developmental disorders and highlighting the novel mechanisms underlying the onset of the diseases.

As an ongoing project, PsyMuKB will be updated regularly with new mutation data and new annotations in the future. For the moment, PsyMuKB focuses on annotating individual mutation at DNA-level impact. With the rapid advance of prediction tools in functional impact on proteins, PsyMuKB will be able to provide information on mutations for sites corresponding to amino acid residues subject to post-translational modification (PTM), such as N-glycosylation, phosphorylation, *etc*., at large scale. Any questions, comments, and suggestions are welcome, which will help future updates. We expect that PsyMuKB will serve as a valuable resource for the research community working on the identification of genetic variation underlying human diseases.

## Availability

PsyMuKB is freely available for academic research users. All data are fully accessible for batch download at http://psymukb.net/Download. Custom scripts used for processing, analyzing, and plotting the data can be found at http://github.com/NENUBioCompute/PsyMuKB.

## Authors’ contributions

GNL conceived and directed the PsyMuKB project. HW and DC designed the architecture. XT, SG, and JW designed and implemented the different modules of the architecture. WW, WS, and WQ curated and processed all of the data for the database. ZW and SY participated in data collection and provided clinical insights on data interpretations. GNL, DC, and HW wrote and edited the manuscript. All authors read and approved the final manuscript.

## Competing interests

The authors have declared no competing interests.

## References

[1] Lynch M. (2010). Rate, molecular spectrum, and consequences of human mutation. Proc Natl Acad Sci U S A.

[2] Roach J.C., Glusman G., Smit A.F.A., Huff C.D., Hubley R., Shannon P.T. (2010). Analysis of genetic inheritance in a family quartet by whole-genome sequencing. Science.

[3] Weischenfeldt J., Symmons O., Spitz F., Korbel J.O. (2013). Phenotypic impact of genomic structural variation: insights from and for human disease. Nat Rev Genet.

[4] Veltman J.A., Brunner H.G. (2012). *De novo* mutations in human genetic disease. Nat Rev Genet.

[5] Werling D.M., Brand H., An J.-Y., Stone M.R., Zhu L., Glessner J.T. (2018). An analytical framework for whole-genome sequence association studies and its implications for autism spectrum disorder. Nat Genet.

[6] Deciphering Developmental Disorders S., McRae J.F., Clayton S., Fitzgerald T.W., Kaplanis J., Prigmore E. (2017). Prevalence and architecture of *de novo* mutations in developmental disorders. Nature.

[7] Turner T.N., Coe B.P., Dickel D.E., Hoekzema K., Nelson B.J., Zody M.C. (2017). Genomic patterns of *de novo* mutation in simplex autism. Cell.

[8] Brandler W.M., Sebat J. (2015). From de novo mutations to personalized therapeutic interventions in autism. Annu Rev Med.

[9] Fromer M., Pocklington A.J., Kavanagh D.H., Williams H.J., Dwyer S., Gormley P. (2014). *De novo* mutations in schizophrenia implicate synaptic networks. Nature.

[10] Yuen R.K.C., Thiruvahindrapuram B., Merico D., Walker S., Tammimies K., Hoang N. (2015). Whole-genome sequencing of quartet families with autism spectrum disorder. Nat Med.

[11] Wang E.T., Sandberg R., Luo S., Khrebtukova I., Zhang L., Mayr C. (2008). Alternative isoform regulation in human tissue transcriptomes. Nature.

[12] Pan Q., Shai O., Lee L.J., Frey J., Blencowe B.J. (2008). Deep surveying of alternative splicing complexity in the human transcriptome by high-throughput sequencing. Nat Genet.

[13] Steijger T., Abril J.F., Engstrom P.G., Kokocinski F., Hubbard T.J., Guigo R. (2013). Assessment of transcript reconstruction methods for RNA-seq. Nat Methods.

[14] Barbosa-Morais N.L., Irimia M., Pan Q., Xiong H.Y., Gueroussov S., Lee L.J. (2012). The evolutionary landscape of alternative splicing in vertebrate species. Science.

[15] Shalek A.K., Satija R., Adiconis X., Gertner R.S., Gaublomme J.T., Raychowdhury R. (2013). Single-cell transcriptomics reveals bimodality in expression and splicing in immune cells. Nature.

[16] Trapnell C., Williams B.A., Pertea G., Mortazavi A., Kwan G., van Baren M.J. (2010). Transcript assembly and quantification by RNA-Seq reveals unannotated transcripts and isoform switching during cell differentiation. Nat Biotechnol.

[17] Raj B., Blencowe B.J. (2015). Alternative splicing in the mammalian nervous system: recent insights into mechanisms and functional roles. Neuron.

[18] Mele M., Ferreira P.G., Reverter F., DeLuca D.S., Monlong J., Sammeth M. (2015). The human transcriptome across tissues and individuals. Science.

[19] Li Q., Lee J.A., Black D.L. (2007). Neuronal regulation of alternative pre-mRNA splicing. Nat Rev Neurosci.

[20] Grabowski P. (2011). Alternative splicing takes shape during neuronal development. Curr Opin Genet Dev.

[21] Wang W., Corominas R., Lin G.N. (2019). *De novo* mutations from whole exome sequencing in neurodevelopmental and psychiatric disorders: from discovery to application. Front Genet.

[22] Wang S., Mandell J.D., Kumar Y., Sun N., Morris M.T., Arbelaez J. (2018). *De novo* sequence and copy number variants are strongly associated with tourette disorder and implicate cell polarity in pathogenesis. Cell Rep.

[23] Acuna-Hidalgo R., Veltman J.A., Hoischen A. (2016). New insights into the generation and role of *de novo* mutations in health and disease. Genome Biol.

[24] Wang K., Li M., Hakonarson H. (2010). ANNOVAR: functional annotation of genetic variants from high-throughput sequencing data. Nucleic Acids Res.

[25] Lek M., Karczewski K.J., Minikel E.V., Samocha K.E., Banks E., Fennell T. (2016). Analysis of protein-coding genetic variation in 60,706 humans. Nature.

[26] Ng P.C., Henikoff S. (2003). SIFT: Predicting amino acid changes that affect protein function. Nucleic Acids Res.

[27] Adzhubei I.A., Schmidt S., Peshkin L., Ramensky V.E., Gerasimova A., Bork P. (2010). A method and server for predicting damaging missense mutations. Nat Methods.

[28] Davydov E.V., Goode D.L., Sirota M., Cooper G.M., Sidow A., Batzoglou S. (2010). Identifying a high fraction of the human genome to be under selective constraint using GERP plus. PLoS Comput Biol.

[29] Kircher M., Witten D.M., Jain P., O'Roak B.J., Cooper G.M., Shendure J. (2014). A general framework for estimating the relative pathogenicity of human genetic variants. Nat Genet.

[30] Huang N., Lee I., Marcotte E.M., Hurles M.E. (2010). Characterising and predicting haploinsufficiency in the human genome. PLoS Genet.

[31] Ardlie K.G., DeLuca D.S., Segre A.V., Sullivan T.J., Young T.R., Gelfand E.T. (2015). The Genotype-Tissue Expression (GTEx) pilot analysis: multitissue gene regulation in humans. Science.

[32] Kang H.J., Kawasawa Y.I., Cheng F., Zhu Y., Xu X., Li M. (2011). Spatio-temporal transcriptome of the human brain. Nature.

[33] Zhong S., Zhang S., Fan X., Wu Q., Yan L., Dong J. (2018). A single-cell RNA-seq survey of the developmental landscape of the human prefrontal cortex. Nature.

[34] Xue Z., Huang K., Cai C., Cai L., Jiang C-y, Feng Y. (2013). Genetic programs in human and mouse early embryos revealed by single-cell RNA sequencing. Nature.

[35] Saunders A., Macosko E.Z., Wysoker A., Goldman M., Krienen F.M., de Rivera H. (2018). Molecular diversity and specializations among the cells of the adult mouse brain. Cell.

[36] Fishilevich S., Nudel R., Rappaport N., Hadar R., Plaschkes I., Stein T.I. (2017). GeneHancer: genome-wide integration of enhancers and target genes in GeneCards. Database.

[37] Lizio M., Harshbarger J., Shimoji H., Severin J., Kasukawa T., Sahin S. (2015). Gateways to the FANTOM5 promoter level mammalian expression atlas. Genome Biol.

[38] Chatr-aryamontri A., Oughtred R., Boucher L., Rust J., Chang C., Kolas N.K. (2017). The BioGRID interaction database: 2017 update. Nucleic Acids Res.

[39] Franz M., Lopes C.T., Huck G., Dong Y., Sumer O., Bader G.D. (2016). Cytoscape.js: a graph theory library for visualisation and analysis. Bioinformatics.

[40] Niknafs N., Kim D., Kim R., Diekhans M., Ryan M., Stenson P.D. (2013). MuPIT interactive: webserver for mapping variant positions to annotated, interactive 3D structures. Hum Genet.

[41] Jonsson H., Sulem P., Kehr B., Kristmundsdottir S., Zink F., Hjartarson E. (2017). Parental influence on human germline *de novo* mutations in 1,548 trios from Iceland. Nature.

[42] Li J., Oehlert J., Snyder M., Stevenson D.K., Shaw G.M. (2017). Fetal *de novo* mutations and preterm birth. PLoS Genet.

[43] Sebat J., Lakshmi B., Malhotra D., Troge J., Lese-Martin C., Walsh T. (2007). Strong association of *de novo* copy number mutations with autism. Science.

[44] Xu B., Roos J.L., Levy S., Van Rensburg E.J., Gogos J.A., Karayiorgou M. (2008). Strong association of *de novo* copy number mutations with sporadic schizophrenia. Nat Genet.

[45] Malhotra D., McCarthy S., Michaelson J.J., Vacic V., Burdick K.E., Yoon S. (2011). High frequencies of *de novo* CNVs in bipolar disorder and schizophrenia. Neuron.

[46] Levy D., Ronennus M., Yamrom B., Lee Y.-H., Leotta A., Kendall J. (2011). Rare *de novo* and transmitted copy-number variation in sutistic spectrum disorders. Neuron.

[47] Gonzalez-Mantilla A.J., Moreno-De-Luca A., Ledbetter D.H., Martin C.L. (2016). A cross-disorder method to identify novel candidate genes for developmental brain disorders. JAMA Psychiatry.

[48] Turner T.N., Yi Q., Krumm N., Huddleston J., Hoekzema K., Stessman H.A.F. (2017). NAR breacthrough article denovo-db: a compendium of human *de novo* variants. Nucleic Acids Res.

[49] Li J., Cai T., Jiang Y., Chen H., He X., Chen C. (2016). Genes with *de novo* mutations are shared by four neuropsychiatric disorders discovered from NPdenovo database. Mol Psychiatry.

[50] Gilissen C., Hehir-Kwa J.Y., Thung D.T., van de Vorst M., van Bon B.W.M., Willemsen M.H. (2014). Genome sequencing identifies major causes of severe intellectual disability. Nature.

[51] Bernier R., Golzio C., Xiong B., Stessman H.A., Coe B.P., Penn O. (2014). Disruptive *CHD8* mutations define a subtype of autism early in development. Cell.

[52] Wilkinson B., Grepo N., Thompson B.L., Kim J., Wang K., Evgrafov O.V. (2015). The autism-associated gene chromodomain helicase DNA-binding protein 8 (*CHD8*) regulates noncoding RNAs and autism-related genes. Transl Psychiatry.

[53] Cotney J., Muhle R.A., Sanders S.J., Liu L., Willsey A.J., Niu W. (2015). The autism-associated chromatin modifier *CHD8* regulates other autism risk genes during human neurodevelopment. Nat Commun.

